# The Role of Right Ventricular Embryology in Echocardiographic Assessment of RV Function

**DOI:** 10.1111/echo.70255

**Published:** 2025-08-04

**Authors:** Ghassan Alnaami

**Affiliations:** ^1^ Family First Medical Clinic Edmonton Alberta Canada

**Keywords:** cardiac development, echocardiography, embryology, morphogenesis, right ventricle, RV function, RV longitudinal strain

## Abstract

The right ventricle (RV) possesses a complex geometry and unique functional characteristics stemming from its embryologic development. Unlike the left ventricle (LV), which arises primarily from the primary heart field, the RV originates predominantly from the anterior or secondary heart field, influencing its structural organization, myocardial fiber orientation, and contractile patterns. This embryologic origin is not only essential for understanding congenital anomalies but also profoundly impacts the interpretation and accuracy of echocardiographic modalities used to assess RV function. This review explores how knowledge of RV embryology enhances the clinical application of echocardiographic methods, including tricuspid annular plane systolic excursion (TAPSE), tissue Doppler imaging (TDI), RV fractional area change (FAC), 3D echocardiography, and RV longitudinal strain. Incorporating embryologic insights provides a more anatomically and physiologically grounded approach to evaluating RV performance, especially in congenital heart disease and right heart failure.

## Graphical abstract

This review highlights how the embryologic segmentation of the right ventricle informs echocardiographic assessment, linking each anatomical compartment to specific imaging modalities. Understanding these developmental foundations enhances the accuracy of RV functional evaluation in both congenital and acquired heart disease.

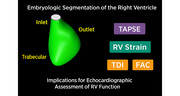

## 1 Introduction

The right ventricle (RV) plays a pivotal role in cardiovascular physiology by serving as the primary pump for the pulmonary circulation. Despite this essential function, the assessment of RV performance remains significantly more complex and nuanced than that of the left ventricle (LV). This complexity arises from several unique anatomical and physiological characteristics of the RV, including its crescentic shape, asymmetric geometry, thin myocardial wall, and relatively lower‐pressure hemodynamic environment. Moreover, RV contraction involves a combination of longitudinal shortening, inward radial motion, and bellows‐like free wall movement, with a high dependence on interventricular interaction and pericardial constraint. These features render conventional imaging methods less reliable and often inadequate for comprehensive RV assessment.

Adding to this complexity is the RV's distinct embryologic origin, which diverges markedly from that of the LV. While the LV develops predominantly from the primary heart field, the RV arises chiefly from the secondary (anterior) heart field—a specialized progenitor cell population that contributes to the right‐sided inflow tract, trabecular myocardium, and outflow tract (conus arteriosus). This embryologic segmentation gives rise to three anatomically and functionally distinct regions within the RV: the inlet portion, the trabecular free wall, and the outlet or conal portion. Each of these segments displays unique myocardial fiber orientation, contractile behavior, and vulnerability to pathological processes. This segmentation has direct implications for congenital heart disease, particularly in conditions affecting the outflow tract and conal structures [1, 2].

Understanding the embryologic underpinnings of RV anatomy and mechanics is essential not only for appreciating its physiological role but also for optimizing imaging strategies. Many commonly used echocardiographic techniques—such as tricuspid annular plane systolic excursion (TAPSE), tissue Doppler imaging (TDI), fractional area change (FAC), 3D echocardiography, and RV longitudinal strain (RVLS)—selectively assess specific embryologic compartments of the RV, influencing their sensitivity and specificity in detecting regional dysfunction. Moreover, congenital heart defects such as tetralogy of Fallot, Ebstein anomaly, and double outlet RV often stem from developmental disruptions in these distinct embryonic regions, further underscoring the relevance of embryology in clinical practice.

This review aims to bridge the gap between developmental biology and clinical cardiology by detailing how the embryological architecture of the RV informs the interpretation and limitations of current echocardiographic modalities. By integrating insights from embryology into routine imaging assessment, clinicians can achieve a more nuanced, compartment‐specific understanding of RV function, thereby improving diagnostic precision, risk stratification, and therapeutic decision‐making.

## 2 Right Ventricular Embryology: An Overview

The development of the human heart begins during the third week of gestation, originating from the splanchnic mesoderm of the cardiogenic region. This mesoderm gives rise to paired endocardial tubes that fuse along the midline to form the primitive linear heart tube. The heart tube is initially composed of several regions arranged from cranial to caudal: the truncus arteriosus, bulbus cordis, primitive ventricle, primitive atrium, and sinus venosus. These segments undergo substantial remodeling and looping to give rise to the mature heart chambers and great vessels.

Around the fourth week of development, the straight heart tube begins to bend to the right, forming a C‐shape and then an S‐shape. This bending helps arrange the heart's parts in the correct positions, making sure the ventricles and outflow tracts line up properly. At the same time, a special group of cells called the secondary heart field (SHF) moves into the front and right side of the heart tube. These cells, guided by specific signals like Isl1, Fgf10, and Tbx1, go on to form the RV, the infundibulum (the smooth part of the RV leading to the pulmonary artery), and parts of the heart's outflow tract.

Structurally, the embryologically derived RV is characterized by longitudinally oriented myocardial fibers, contrasting with the circumferential and oblique fibers of the LV. This fiber arrangement underlies the RV's characteristic longitudinal contraction during systole, which influences the selection of echocardiographic methods sensitive to this type of motion. Unlike the LV, which thickens radially, the RV primarily contracts by shortening along its longitudinal axis, a feature rooted in its embryonic lineage.

The maturing RV can be subdivided into three embryologically and functionally distinct regions (Table 1):

- Inlet (Sinus) portion: This region originates from the atrioventricular canal and receives venous blood from the right atrium through the tricuspid valve. It plays a significant role in diastolic filling and is structurally associated with the septal and anterior tricuspid valve leaflets. The inlet contributes to annular motion and participates in the early stages of RV diastolic relaxation.
- Trabecular portion: This constitutes the largest portion of the RV and arises from the proximal bulbus cordis. It comprises the heavily trabeculated free wall and apex, populated by longitudinally oriented myofibers. The trabecular segment serves as the principal contractile engine of the RV and is the primary contributor to echocardiographic indices that measure longitudinal function, such as TAPSE and RVLS.
- Outlet (Conal) portion: Also known as the infundibulum, this region is derived from the conus arteriosus of the SHF. It forms the smooth‐walled outflow tract leading to the pulmonary valve and is essential for the spatial alignment and septation of the aortic and pulmonary outflows. Its muscular wall contains fibers arranged in more oblique and radial patterns, contributing uniquely to systolic ejection. Importantly, this region is often underrepresented in traditional two‐dimensional echocardiographic views.

**TABLE 1:** The anatomical parts of the right ventricle (RV), their embryological origins, anatomical locations, and primary functional contributions.

RV part	Embryological origin	Anatomical location	Primary function
Inlet (Sinus) portion	Sinus venosus/atrioventricular canal	Posterobasal region; supports tricuspid valve	Receives systemic venous return and guides diastolic inflow
Trabecular portion	Bulbus cordis (muscularized)	Mid to apical free wall	Generates systolic force via longitudinal contraction
Outlet (Conal) portion	Conus arteriosus/secondary heart field	Anterosuperior RV leading to pulmonary valve	Facilitates smooth systolic ejection into the pulmonary artery

Each region's developmental pathway and resultant myoarchitecture directly influence its mechanical behavior. The RV's systolic performance is dominated by longitudinal shortening, primarily driven by contraction of the trabecular and inlet regions, while the outlet portion contributes less to longitudinal motion but is essential for coordinated ejection. Consequently, echocardiographic modalities that measure longitudinal motion—such as TAPSE, TDI, and RV strain—are highly sensitive to changes in the inlet and trabecular segments. In contrast, modalities like 3D echocardiography and cardiac MRI offer the advantage of assessing global RV performance, including the often‐overlooked outlet component.

Understanding the embryologic origins of the RV not only illuminates the anatomic basis for regional contraction patterns but also provides a framework for diagnosing and managing congenital anomalies such as tetralogy of Fallot (involving the conal septum), double outlet RV, and Ebstein anomaly (affecting the inlet portion). Moreover, it enhances the interpretation of echocardiographic findings in acquired diseases such as pulmonary hypertension and RV cardiomyopathies, where dysfunction may preferentially affect specific embryologic compartments. As such, knowledge of RV embryology is fundamental for accurate echocardiographic assessment and clinical decision‐making [1, 2, 3, 4, 5, 6].

**FIGURE 1:**
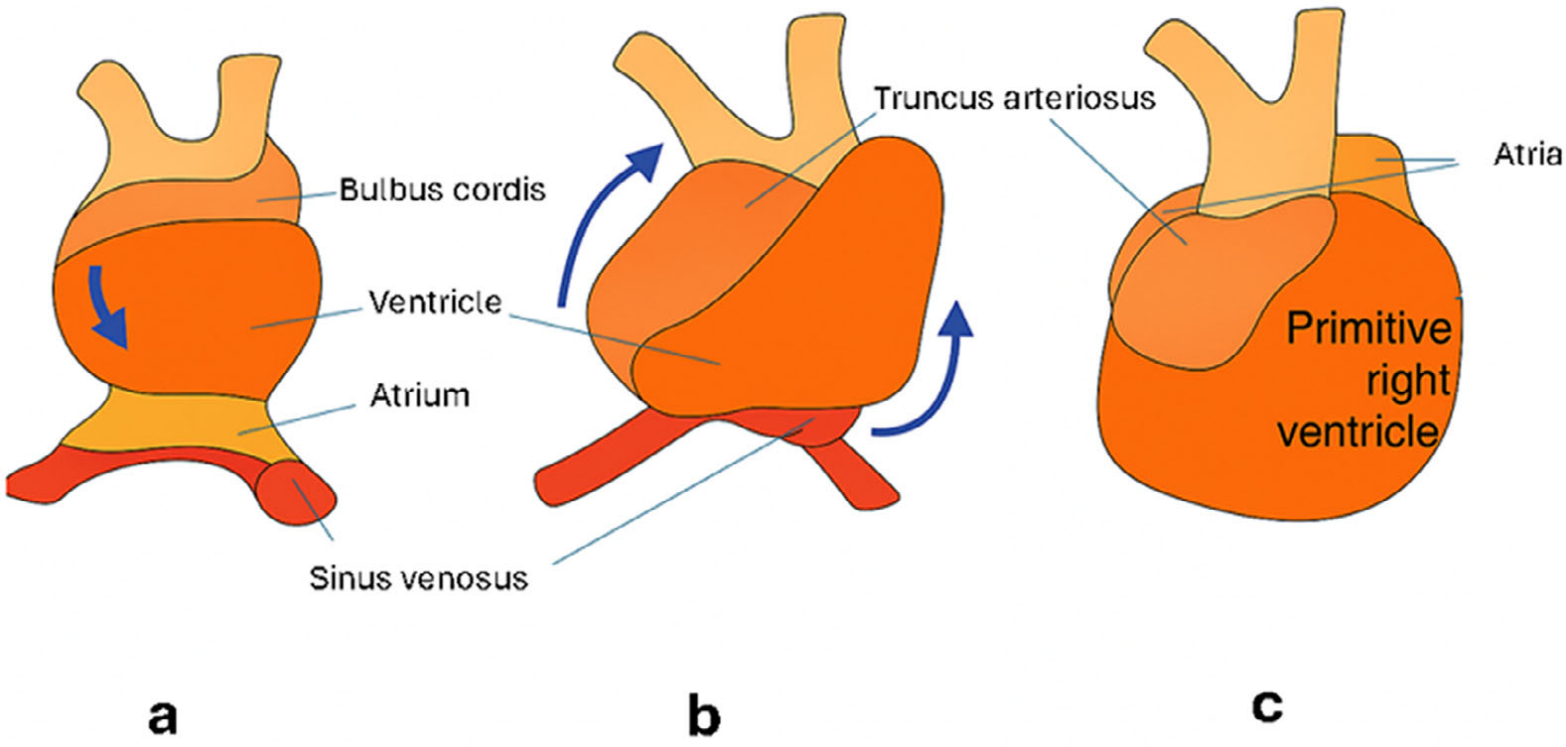
Sequential development of the embryonic heart.

Figure 1 illustrates the sequential stages of early heart development, highlighting the emergence and positioning of the RV through three key morphogenetic transformations: (a) the linear heart tube (Day 22 post conception), (b) the C‐shaped cardiac tube (Day 25), and (c) the S‐shaped cardiac tube (Day 28). In panel (a), the embryonic heart appears as a straight tube composed of distinct segments: the aortic root, bulbus cordis, ventricle, atrium, and sinus venosus, arranged from cranial to caudal ends. The bulbus cordis, which will later contribute to the RV and outflow tract, is located just below the aortic root. As shown in panel (b), the heart tube undergoes rightward looping, forming a C‐shaped structure, which spatially reorients the chambers. This looping initiates the formation of the primitive RV, derived primarily from the bulbus cordis. In panel (c), the heart reaches the S‐shaped configuration, a critical milestone in cardiac morphogenesis. Here, the primitive RV is more distinctly developed and positioned anteriorly and inferiorly, relative to the LV. This transformation lays the foundational architecture for chamber‐specific growth and functional specialization. The labeled arrows in each panel illustrate the direction of looping and chamber repositioning that contribute to the heart's final anatomical configuration.

## 3 Embryologic Insights Informing Echocardiographic Methods

Table 2 summarizes key echocardiographic modalities used to assess right ventricular (RV) function, distinguishing between systolic and diastolic parameters. Systolic function is primarily evaluated using TAPSE, tissue Doppler systolic velocity (S′), FAC, three‐dimensional (3D) echocardiographic ejection fraction, and right ventricular longitudinal strain (RVLS). These modalities predominantly reflect the contractile performance of the inlet and trabecular portions of the RV, which arise from the secondary heart field and are characterized by longitudinal fiber orientation. Diastolic function, on the other hand, is assessed using TDI (E′, A′), pulsed‐wave Doppler of tricuspid inflow (E/A ratio), hepatic vein flow patterns, and isovolumic relaxation time (IVRT). These indices offer insights into RV compliance, relaxation, and filling dynamics, complementing the systolic evaluation and providing a comprehensive assessment of RV performance.

**TABLE 2:** Echocardiographic assessment of right ventricular function.

Modality	Parameter measured	Function assessed	Comments/embryologic focus
TAPSE	Longitudinal annular displacement (M‐mode)	Systolic	Reflects contraction of the inlet and trabecular segments derived from SHF; simple and reproducible.
TDI S′ wave	Peak systolic velocity of the lateral tricuspid annulus	Systolic	Focuses on longitudinal fibers of trabecular myocardium; angle‐dependent.
RV FAC	Area change between diastole and systole (2D)	Systolic	Reflects global RV function; influenced by trabecular and partially outlet contraction.
3D echocardiography	RV end‐diastolic and end‐systolic volumes, EF	Systolic (Global)	Evaluates entire RV, including conal (outlet); overcomes geometric limitations of 2D.
RV longitudinal strain (RVLS)	Deformation of RV free wall segments (speckle tracking)	Systolic	Highly sensitive to trabecular function; an early marker of systolic dysfunction.
TDI E′ and A′ waves	Early and late diastolic velocities of the tricuspid annulus	Diastolic	Evaluates relaxation and atrial contribution to RV filling.
Tricuspid inflow (PW Doppler)	E and A waves through the tricuspid valve	Diastolic	E/A ratio and deceleration time provide estimates of RV compliance and filling pressures.
Hepatic vein Doppler	Venous flow pattern into the right atrium	Diastolic	Assesses RV compliance and right atrial pressure via reversal patterns.
TDI IVRT	Isovolumic relaxation time	Diastolic	Prolonged in impaired relaxation; measured using tissue Doppler.

### 3.1 TAPSE

**FIGURE 2:**
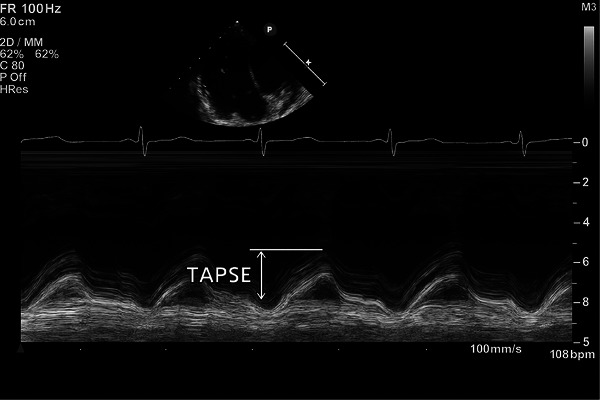
Quantification of TAPSE via m‐mode at the lateral tricuspid annulus.

TAPSE is a linear M‐mode measurement that quantifies the longitudinal displacement of the lateral tricuspid annulus during systole (Figure 2). As the RV contracts, particularly in the basal regions, the annulus moves toward the apex, and TAPSE captures this motion. Normal values are listed in Table 3. The ASE guidelines recommend TAPSE as a standard linear parameter for RV systolic function in adults [4].

**TABLE 3:** Reference values and clinical interpretation of TAPSE [7, 8].

Population	TAPSE value	Interpretation
Adults	≥17 mm	Normal RV systolic function
	<17 mm	Suggestive of RV systolic dysfunction
Neonates	∼6–10 mm	Age‐appropriate normal range
Children (5–10 y)	∼13–17 mm	Age‐appropriate normal range
Adolescents	≥17 mm	Approaching adult normal range

Embryologically, this contraction reflects the activity of the inlet and trabecular components derived from the atrioventricular canal and bulbus cordis. TAPSE has been validated as a predictor of prognosis in heart failure, pulmonary hypertension, and congenital heart disease. Its advantages include ease of acquisition, high reproducibility, and strong correlation with RV ejection fraction. However, TAPSE only assesses longitudinal function, does not reflect global or segmental dysfunction outside the lateral wall, and is load‐dependent. Importantly, it ignores the contractile behavior of the conal region and may underestimate dysfunction in diseases affecting the RV outflow tract [1, 3, 4, 5, 7, 8].

### 3.2 TDI

TDI measures myocardial velocity in specific regions, particularly focusing on the systolic (S′) velocity of the lateral tricuspid annulus. The annulus represents the functional output of the underlying embryologic compartments—primarily the inlet and trabecular zones. This velocity‐based modality can detect impaired longitudinal function even before global measures decline. TDI has demonstrated sensitivity in detecting subclinical dysfunction in various conditions, including systemic sclerosis, chemotherapy‐induced cardiotoxicity, and congenital heart anomalies. Normal values for different TDI waves have been established (Table 4 and Table 5). Although it shares the longitudinal limitation of TAPSE and is highly angle‐dependent, it offers superior temporal resolution and is well‐suited for serial monitoring. Its inability to visualize or quantify the conal segment, which originates from the secondary heart field, remains a significant limitation in congenital or arrhythmogenic RV diseases [1, 3, 4, 9].

**TABLE 4:** Normal values for right ventricular TDI parameters in adults (lateral tricuspid annulus) [8, 10, 11, 12].

Parameter	Symbol	Normal value (Adults)	Interpretation
Systolic velocity	S′	≥9.5 cm/s	Normal RV systolic function
Early diastolic velocity	E′	Variable, typically>6–7 cm/s	Assesses RV diastolic relaxation
Late diastolic velocity	A′	Variable, age‐dependent	Reflects the atrial contraction contribution
Isovolumic contraction velocity	IVV	No fixed cutoff; qualitatively assessed	Reflects pre‐ejection myocardial contractility
Myocardial performance index	Tei index	≤0.40 (TDI‐derived)	Normal global RV performance

**TABLE 5:** Pediatric reference values for right ventricular TDI parameters [10, 11, 12].

Age group	S′ (cm/s)	E′ (cm/s)	A′ (cm/s)	Tei index (TDI‐derived)
Neonates (0–1 mo)	6.0–8.5	7.0–10.0	5.0–7.0	0.35–0.45
Infants (1–12 mo)	7.0–10.0	8.0–11.0	6.0–8.0	0.30–0.40
Toddlers (1–3 yrs)	9.0–11.5	9.0–12.0	6.0–8.5	0.28–0.38
Children (4–10 yrs)	10.0–12.5	9.5–12.5	6.5–9.0	0.25–0.35
Adolescents (11–18 yrs)	≥11.0	≥10.0	≥7.0	0.25–0.32

**FIGURE 3:**
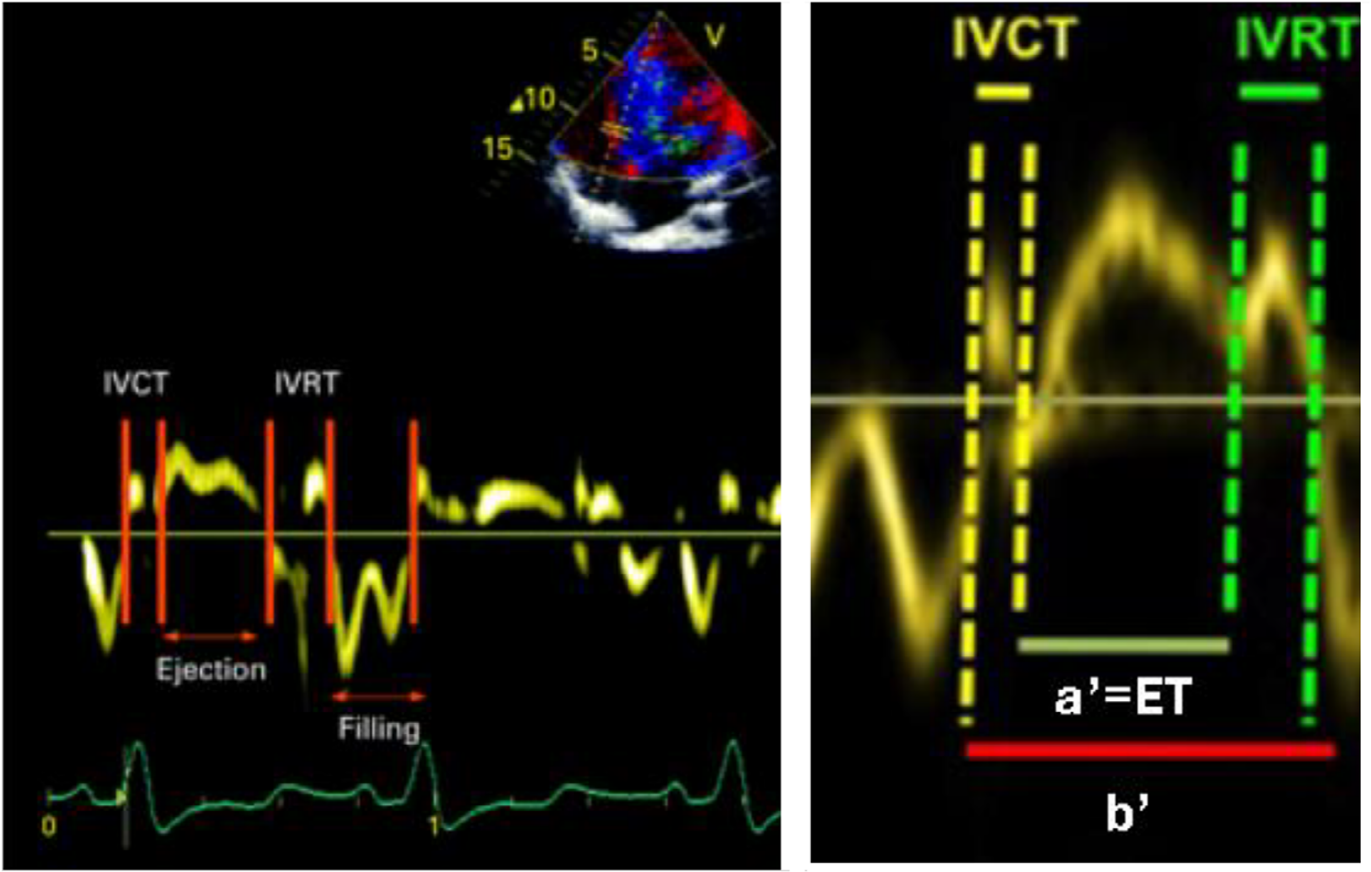
Tissue Doppler imaging (TDI) of the basal segment of the right ventricular free wall. Calculation can be done using the time intervals (left) or an alternative way(right).

Figure 3 displays key myocardial velocity waveforms acquired over a single cardiac cycle, including the systolic (S′) wave, early diastolic (E′) and late diastolic (A′) waves, and the isovolumic contraction velocity (IVCT). These parameters provide comprehensive insight into both systolic and diastolic right ventricular function and contribute to the calculation of the TDI‐derived myocardial performance (Tei) index.

RV index of myocardial performance (RIMP), also known as Tei index, can be obtained by TDI as described earlier. Also, it can be measured using pulsed Doppler echocardiography. Here are the formulas to calculate RIMP.

A. Pulsed‐wave Doppler method:

Formula:

$$\mathrm{Tei}\;\mathrm{Index}=\frac{\mathrm{IVCT}+\mathrm{IVRT}}{\mathrm{ET}}$$

IVCT: isovolumic contraction time, IVRT: isovolumic relaxation time, ET: ejection time

Alternative calculation (using time intervals):

$$\mathrm{Tei}\;\mathrm{Index}=\frac{a-b}{b}$$

- a = time interval between cessation and onset of tricuspid inflow (measured from tricuspid inflow Doppler)
- b = RV ejection time (measured from pulmonary outflow Doppler)

B. TDI method (Figure 3):

Formula:

$$\mathrm{Tei}\;\mathrm{Index}\;\left(\mathrm{TDI}\right)=\frac{\mathrm{IVCT}+\mathrm{IVRT}}{\mathrm{ET}}$$

Alternative calculation (using annular motion):

$$\mathrm{Tei}\;\mathrm{Index}\;\left(\mathrm{TDI}\right)=\frac{a^{\prime }-b^{\prime }}{b^{\prime }}$$

- a′ = time interval between the end and onset of systolic (S′) wave—that is, the total duration of one cardiac cycle (including isovolumic phases)
- b′ = duration of the S′ wave (RV ejection time)

Lower values indicate better RV performance. Normal values are generally **<0.40** for healthy individuals using TDI.

### 3.3 RV FAC

**FIGURE 4:**
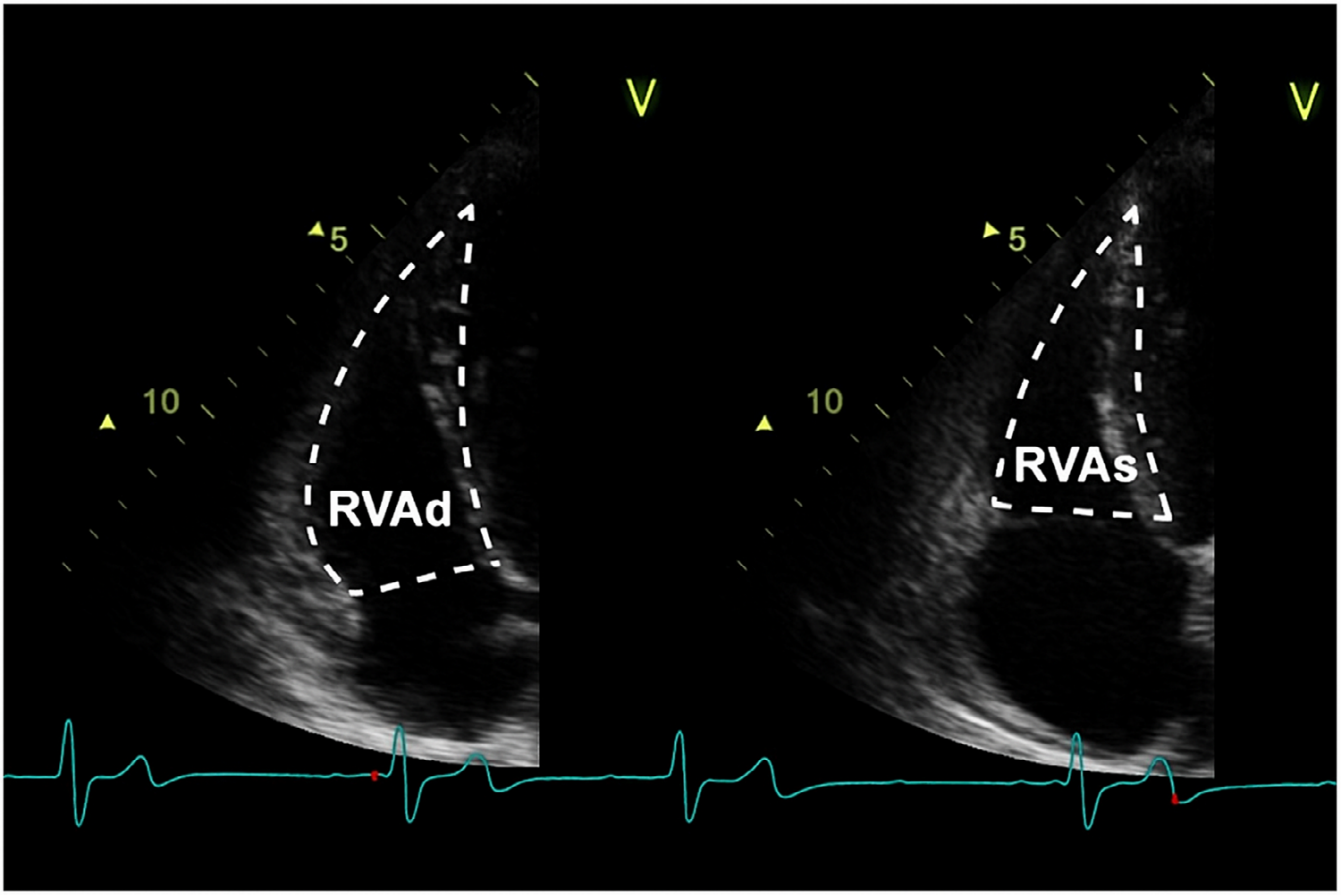
Tracing RV endocardium during systole and diastole to measure FAC. Adapted from: Rudski et al. [8]. RVAd = right ventricular area in diastole, RVAs = right ventricular area in systole

RV FAC is a 2D echocardiographic method calculated as the percentage change in RV end‐diastolic and end‐systolic area, typically in the apical four‐chamber view (Figure 4). It provides a geometric representation of RV systolic function and integrates motion across the inlet, trabecular, and partially the outlet portions. Embryologically, it reflects the contractile contribution of the muscularized bulbus cordis (trabecular segment) and the AV canal (inlet region). Though widely used in clinical settings and part of echocardiographic guidelines (Table 6), FAC is limited by image quality, difficulty in defining RV endocardial borders, and the inability to comprehensively visualize the outflow tract. FAC is also load‐dependent and does not provide insights into regional dysfunction. Nonetheless, its semi‐global scope makes it superior to TAPSE and TDI in representing the RV's composite function [4, 13, 14, 15, 16, 17].

$$\mathrm{FAC}\;\left(\%\right)=\frac{\mathrm{RVAd}-\mathrm{RVAs}}{\mathrm{RVAd}}\times 100$$

**TABLE 6:** Normal values for right ventricular fractional area change (FAC) [7, 8].

Population	RV FAC (%)	Interpretation
Adults	**≥35%**	Normal RV systolic function
	**<35%**	Suggestive of RV systolic dysfunction
Children (BSA‐dependent)	**≥35%–45%** *(variable by age and size)*	Normal RV systolic function in pediatric populations
	**<35%**	May indicate impaired RV function

### 3.4 3D Echocardiography

Three‐dimensional echocardiography has revolutionized RV imaging by allowing volumetric quantification without relying on geometric assumptions. It is capable of measuring RV end‐diastolic and end‐systolic volumes, ejection fraction, and regional wall motion with high accuracy. From an embryologic standpoint, 3D echo is the only modality that fully captures contributions from all three compartments: inlet, trabecular, and conal. This makes it indispensable in the assessment of congenital heart disease, arrhythmogenic RV cardiomyopathy, and postsurgical RV remodeling. In pediatric patients, 3D echocardiography requires tailored acquisition techniques and reference ranges, as RV geometry and function evolve with age and somatic growth [11]. 3D echo has shown a strong correlation with MRI‐derived RV volumes and function. Despite requiring good image quality and technical expertise, it remains the most comprehensive echocardiographic tool for global and regional RV function, overcoming the limitations of 2D views that often exclude the anterior and outflow walls [7, 8, 9, 13, 17, 18].

**FIGURE 5:**
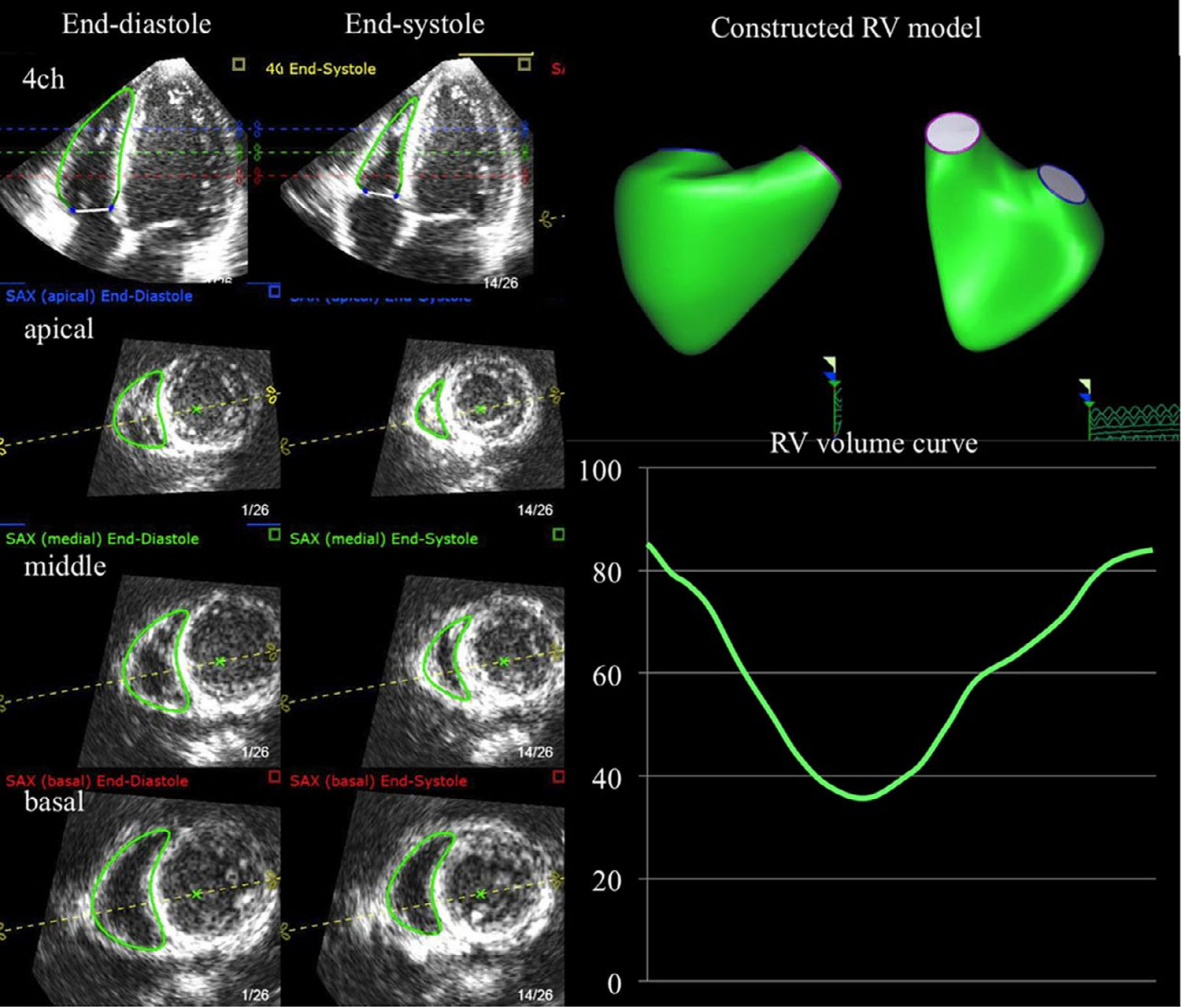
3D Echocardiographic assessment of right ventricular volumes and function.

Figure 5 shows a multiplanar reconstruction from a 3D echocardiographic dataset (TomTec 4D RV Function 2.0 software in this example), demonstrating the RV at end‐diastole and end‐systole across apical, mid, and basal levels. A 3D‐rendered RV model is shown alongside the derived RV volume‐time curve. This technique enables accurate quantification of RV end‐diastolic volume (RVEDV), end‐systolic volume (RVESV), and ejection fraction (RVEF) without geometric assumptions, allowing comprehensive evaluation of global and regional RV function—including the conal (outlet) portion often underrepresented in 2D imaging. Table 7 shows the normal 3D values for RV function.

**TABLE 7:** Normal 3D echocardiographic values for right ventricular (RV) function [7, 18].

Parameter	Adult	Children
RV end‐diastolic volume (RVEDV)	Men: <114 mL Women: <102 mL	< 74 mL/m^2^ Neonates: 20–30 mL Adolescents: 70–100 mL
RV end‐systolic volume (RVESV)	Men: <65 mL Women: <54 mL	< 44 mL/m^2^ Neonates: 10–15 mL Adolescents: 30–50 mL
RV ejection fraction (RVEF)	Normal: ≥45% Borderline: 40%–44% Abnormal: <40%	Normal: ≥50%–55%

### 3.5 RVLS

RVLS, derived from speckle‐tracking echocardiography, quantifies myocardial deformation as a percentage of longitudinal fiber shortening. It is particularly sensitive to subclinical dysfunction and is a robust predictor of adverse outcomes in pulmonary hypertension, chronic lung disease, and systemic inflammatory disorders. The embryologic relevance of RVLS lies in its assessment of the mid and apical segments of the free wall—regions formed from the muscularized bulbus cordis. It excludes septal segments influenced by LV function, thus offering RV‐specific insights. However, RVLS still neglects the conal portion and is influenced by image quality and software variability. Despite these limitations, RVLS has become an essential parameter in modern RV assessment, combining sensitivity, prognostic value, and correlation with advanced imaging modalities [9, 16, 19, 20, 21, 22, 23].

**FIGURE 6:**
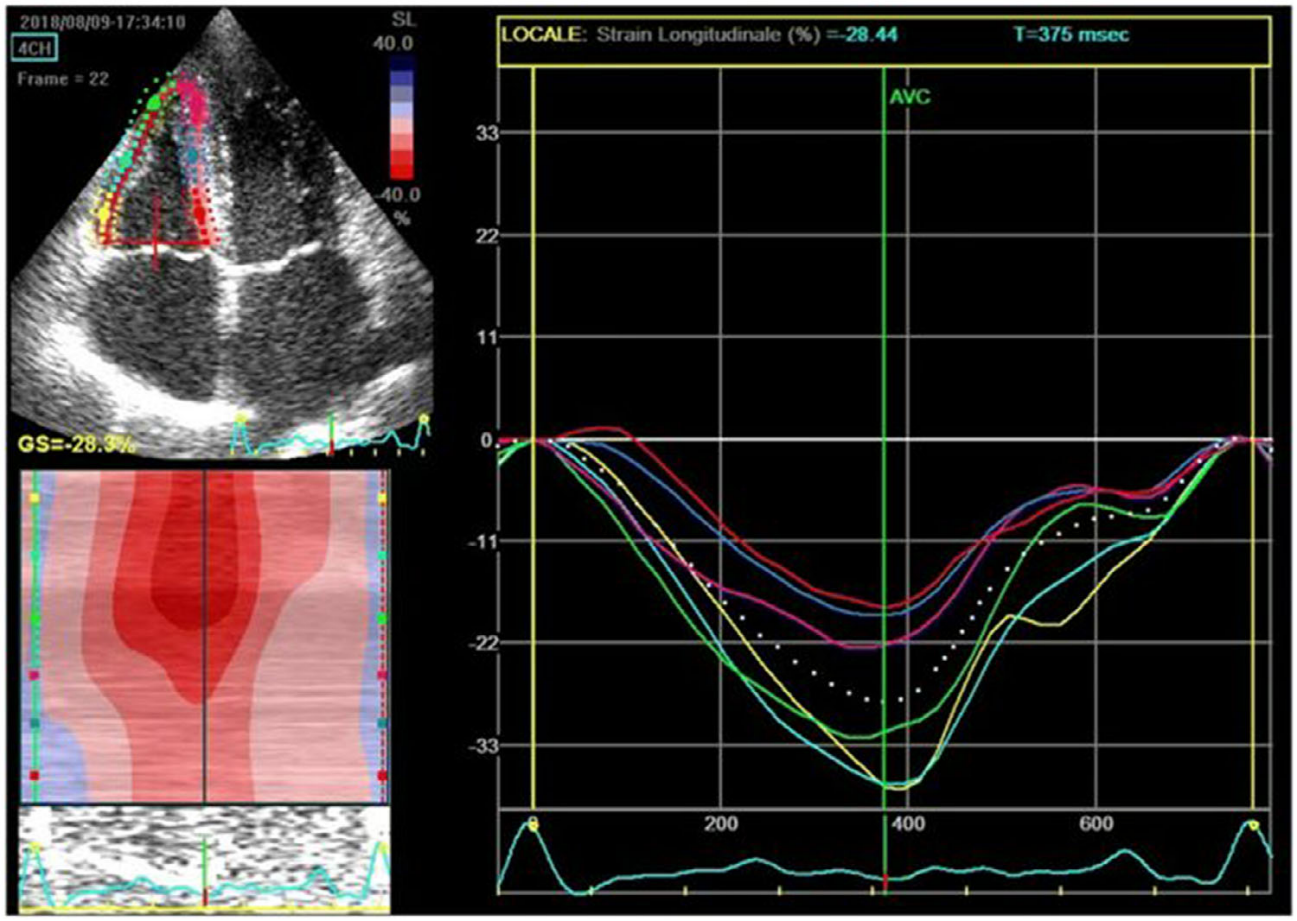
Global right ventricular longitudinal strain (RVLS) Measurement using speckle‐tracking echocardiography analysis.

Figure 6 demonstrates assessment of RVLS performed using a focused apical four‐chamber view that includes the entire right ventricular free wall and interventricular septum. The endocardial border is manually traced using a point‐and‐click method to generate a region of interest divided into six segments—three along the RV free wall and three along the interventricular septum. The average peak systolic longitudinal deformation across these six segments is calculated to derive the global RVLS, providing a sensitive measure of RV systolic function. Several software programs are available to the user. EchoPAC by GE Healthcare was used in this figure.

Normal values for right ventricular (RV) longitudinal strain and strain rate (SR, Table 8) differ between adults and children due to developmental and hemodynamic factors. In healthy adults, global RV free wall longitudinal strain typically ranges from −20% to −30%, with values less negative than −20% suggesting impaired systolic function. In children, especially infants and younger age groups, RV strain values are generally more negative—−25% to −35%—reflecting higher myocardial compliance and contractility. Similarly, SR values are higher in children, with systolic SRs ranging from −2.0 to −3.0 s^−1^, compared to −1.5 to −2.5 s^−1^ in adults. These differences emphasize the importance of age‐ and body‐size–adjusted reference values, particularly in pediatric assessments.

**TABLE 8:** Normal RV longitudinal strain and strain rate values (adults and children) [15, 16].

Parameter	Adults	Children
Global RV free wall strain (RVLS) (>−20% suggests dysfunction)	−20% to −30%	−25% to −35% More negative in neonates/infants; decreases slightly with age
RV septal strain	−15% to −20%	−20% to −25% (varies by age and loading)
Global RV strain (6‐segment average)	−18% to −25%	−22% to −30%
RV systolic strain rate (SRs)	−1.5 to −2.5 s^−1^	−2.0 to −3.0 s^−1^ (higher in younger children)
RV early diastolic SR (SRe)	1.5 to 2.5 s^−1^	2.0 to 3.0 s^−1^
RV late diastolic SR (SRa)	0.8 to 1.5 s^−1^	1.0 to 2.0 s^−1^

*Note*: RV Free Wall Strain is preferred over global RV strain (which includes septal segments influenced by LV function). More negative values indicate better myocardial deformation. Pediatric values are generally more negative than adult values due to higher heart rates and myocardial compliance. Reference ranges vary slightly depending on vendor‐specific software, imaging frame rate, and tracking algorithm.

While several published studies discuss aspects of RV anatomy, embryology, or echocardiographic evaluation separately, none seem to combine these topics to provide a unified framework for understanding how embryologic development informs echocardiographic assessment of RV function. These articles, while informative, do not provide the integrative perspective that your manuscript offers [24, 25]. Therefore, this article appears to be novel in its approach to linking RV embryology with echocardiographic assessment, potentially filling a gap in the current literature, and integrates right ventricular (RV) embryology with echocardiographic assessment (Table 9).

**TABLE 9:** Echocardiographic methods and embryological basis in RV function.

Method	Assessed motion	Primary embryologic segments evaluated	Strengths	Limitations
TAPSE (tricuspid annular plane systolic excursion)	Longitudinal displacement of the lateral tricuspid annulus	Inlet (atrioventricular canal) and Trabecular (bulbus cordis)	Simple, reproducible; reflects dominant longitudinal motion	Does **not** assess the outlet (conal) portion; angle‐ and position‐dependent
TDI (tissue Doppler imaging)	Longitudinal systolic velocity at the tricuspid annulus	Inlet and Trabecular	Sensitive to early dysfunction; velocity‐based (not just distance)	Angle‐dependent; misses conal/outlet dysfunction
RV FAC (fractional area change)	Global area change between systole and diastole	Primarily Trabecular; partial Inlet and Conal (if visible)	Composite index; captures radial and longitudinal change	Limited conal visualization; dependent on image quality and chamber geometry
3D echocardiography	Volumetric contraction (RVEF); regional wall motion	All: Inlet, Trabecular, and Outlet (conus arteriosus)	Captures full RV geometry and function; includes outlet	Less accessible; operator and software dependent; lower temporal resolution
RV longitudinal strain (RVLS)	Longitudinal myocardial deformation (free wall)	Primarily Trabecular; part of Inlet	Highly sensitive to early systolic dysfunction; tracks fiber deformation	Excludes Outlet portion; requires good image quality and strain tracking software

## 4 Conclusion

The RV's structure and function are deeply rooted in its embryologic origins, which give rise to its three morphologically distinct components—the inlet, trabecular, and outlet portions. Each of these segments demonstrates unique contractile properties and responds differently to hemodynamic stress and pathological conditions. As a result, no single echocardiographic modality can comprehensively evaluate the entirety of RV function. Instead, accurate and clinically meaningful assessment necessitates a nuanced, segmental approach rooted in developmental anatomy.

TAPSE and TDI effectively assess the longitudinal motion derived from the inlet and trabecular components, offering sensitive early detection of dysfunction in these regions. However, they fail to evaluate the outlet portion, which has distinct embryologic and mechanical properties. RV FAC provides a more integrated assessment but remains limited by geometric assumptions and visual access to the outflow tract. In contrast, 3D echocardiography overcomes these limitations by allowing for full‐chamber evaluation and quantification of regional function across all embryologic compartments. RVLS provides detailed information on myocardial deformation in the free wall, again reflecting primarily the trabecular contribution to systolic function.

By incorporating the principles of cardiac embryology into echocardiographic interpretation, clinicians can tailor their diagnostic strategies to reflect the underlying anatomical and functional heterogeneity of the RV. This is particularly critical in the evaluation of congenital heart diseases and in the early detection of acquired conditions affecting specific regions of the RV. Ultimately, an embryologically‐informed approach to RV imaging enhances our understanding of disease mechanisms, improves diagnostic accuracy, and supports individualized patient management.

Future research should continue to refine imaging techniques with attention to the embryologic segmentation of the RV, potentially enabling the development of new indices that better quantify regional dysfunction. Such advances will pave the way for more precise phenotyping of right heart disease and targeted therapeutic interventions.

## Data Availability

The data that support the findings of this study are openly available at https://doi.org/10.1017/S1047951110001150, reference number 10.1017/S1047951110001150.
